# Office Card

**Published:** 1879-07

**Authors:** 


OFFICE CARD.—Dr. H V. HICKS & Co., Preston, Iowa, send us a very ingeniously constructed card for leaving directions in Physicians' offices when absent. It fully meets almost every situation, and would be very convenient for physicians who do not keep a constant attendant in their offices.

